# Supplementary material for: Sensing of viral and endogenous RNA by ZBP1/DAI induces necroptosis
Source: EMBO J. 2017 Jul 17;36(17):2529–43. doi: 10.15252/embj.201796476 (PMC5579359; doi:10.15252/embj.201796476)
Supplement: Supplementary file 1 — Appendix [file EMBJ-36-2529-s001.pdf]

Appendix:

Appendix Supplementary Methods 1

Appendix Table S1: Primers for genotyping and cloning 6

Appendix Table S2: Primers and probes for (RT)-qPCR 7

Appendix Table S3: Antibodies used for Western blot 8

Appendix Table S4: siRNA sequences 9

Appendix Supplementary Methods

ZBP1-Zα1α2mut transgenic mice

5' and 3' homology arms were PCR amplified from C57BL/6 Bac clones RP24-289A19 and RP24-299I12. The N46A (AAT to GCT) and Y50A (TAC to GCC) mutations were introduced into exon 2 in the 5' homology arm. The N122A (AAC to GCC) and Y126A (TAT to GCT) mutations were introduced into exon 3 in the 3' homology arm. The homology arms were cloned into the targeting vector using AscI and PspXI (5' homology arm) and KpnI and NotI (3' homology arm) restriction sites. DTA cloned upstream of the 5' homology arm was used for negative selection. A Neo cassette is inserted between the homology arms and is flanked by two LoxP-sites. The Neo cassette contains a Cre-recombinase gene driven by the testes specific promotor of angiotensin converting enzyme, which self-deletes the cassette when going through male germline (Bunting *et al*, 1999). The linearized vector was electroporated into ES cells (C57Bl/6), followed by drug selection, PCR screening, and Southern Blot confirmation. Out of 139 drug-resistant clones, 6 were confirmed positive for the targeting construct. Two clones were

selected for blastocyst injection, followed by chimera production. Four heterozygous founder males from each ES-cell line were selected for breeding to C57Bl/6 females to generate Neo-deleted *Zbp1*$^{+/Z_{\alpha}1_{\alpha}2}$ offspring. Sequencing and genotyping primers are listed in Appendix Table S1. ZBP1-Zα1α2 primary MEFs were isolated from E12.5-14.5 embryos from heterozygous breeding pairs.

<u>Virus production and infection</u>

Cells were infected with a MOI of 0.001 and 2 hours later the inoculum was washed away. Virus was harvested 2 days after 100% CPE was reached. Virus was concentrated by centrifugation at 30,000 g for 1 hour and viral stocks were sonicated before use. Viral titres were determined by plaque assay on immortalised Zbp1-/- MEFs. Cells were infected for 2 hours in DMEM containing 10% FCS, after which the viral inoculum was washed away and fresh medium was added to the cells. For measuring viral genome replication, MCMV was incubated with 10 U/ml DNase I (Turbo DNase I, ThermoFisher Scientific) for 30 min at 37□C in 1X reaction buffer diluted in PBS. 2 hours after infection with DNase-treated virus, cells were washed several times to remove the inoculum and fresh medium was provided. 50 µM ganciclovir (GCV, Merck) was added to the cells at the time of infection. MCMV was UV inactivated by exposing to 250 mJ/cm2 UV-C (254nm; Spectrolinker XL-1500, Spectroline). For *in vivo* infections, 8-12 week old sex-matched mice were inoculated with $2x10^6$ pfu MCMV-M45^{WT} or MCMV-M45^{mutRHIM} by intraperitoneal injection. 5 days post infection, mice were sacrificed and spleens were harvested and homogenised with glass beads (Sigma Aldrich) in 1 ml DMEM containing 10% FCS on a FastPrep F120 instrument (Thermo Fisher). Debris was removed by centrifugation (300 g, 5 min) and virus-containing supernatant was titrated in duplicate on primary MEFs.

(PAR)-CLIP

7.5 x 10^6 NIH3T3 cells expressing wild type or mutant ZBP1 were seeded into 15 cm diameter tissue culture dishes and one plate per sample was used as input. For PAR-CLIP, cells were treated with 4-thiouridine (Sigma Aldrich) or 6-thioguanosine (Carbosynth) at the time of MCMV-M45^{mutRHIM} infection (MOI 3). 8 hour later cells were washed, overlaid with 5 ml PBS and crosslinked with 150 mJ/cm^2 UV-A (365 nm; Stratalinker 2400, Stratagene) for PAR-CLIP or 150 mJ/cm^2 UV-C (254 nm; Spectrolinker XL-1500, Spectroline) for CLIP. Cells were lysed for 20 min. in 1 ml RIPA buffer (50 mM Tris.HCl pH 7.5, 150 mM NaCl, 1% NP-40, 0.1% SDS, 0.5% deoxycholate). Lysates were incubated for 5 min. at 37°C on a shaker (1100 rpm) with Turbo DNase I (Ambion) followed by a 5 min. incubation with RNase A (Affymetrix) at 37°C at 1100 rpm. Lysates were transferred to ice and RNase A was inactivated with 12.5 µl RNasin Plus (Promega). Lysates were centrifuged for 20 min at 17,000 g and supernatant was used as input. Samples were immunoprecipitated with 6 µg of anti-FLAG (Sigma Aldrich, clone M2) covalently linked to magnetic beads (Dynabeads antibody coupling kit, Life Technologies, 30 µg anti-FLAG per mg beads). After 2 hours, beads were washed twice with high salt wash buffer (50 mM Tris.HCl pH 7.5, 1 M NaCl, 1% NP-40, 0.1% SDS, 0.5% deoxycholate) and once with PNK buffer (20 mM Tris.HCl pH 7.5, 10 mM $MgCl_2$, 0.2% Tween-20). The beads were incubated for 3 min. at 65°C on a shaker (1100 rpm) with 100 µl urea cracking buffer (50 mM Tris.HCl pH 7.5, 6 M Urea, 1% SDS, 25% PBS) to denature RNA-protein complexes and to remove contaminating protein-RNA complexes. Supernatant was separated from the beads and transferred to 1 ml of T-20 buffer (50 mM Tris.HCl pH 7.5, 150 mM NaCl, 0.5% Tween-20, 0.1 mM EDTA). The protein-RNA complexes were immunoprecipitated a second time using 5 µg of

anti-FLAG (Sigma Aldrich, clone M2) coupled to protein G magnetic beads (Dynabeads, Life Technologies). After 2 hours, beads were washed twice with high salt wash buffer and twice with PNK wash buffer. RNA was the labelled on-bead with T4 PNK (NEB) and in 40 μl reaction volume (1 μl T4 PNK, 4 μl 10X PNK buffer, 0.5 μl RNasin Plus, 1 μl γ-32P-ATP; Hartmann Analytic) for 5 min at 37°C at 1100 rpm. The T4 PNK reaction mix was removed and the ZBP1-RNA complexes were eluted from the beads by incubating for 10 min. at 70°C in 20 μl NuPAGE LDS sample buffer (Life Technologies) with 50 mM DTT. Samples were loaded on 4-12% Bis-Tris SDS-PAGE gels (Life Technologies) and transferred on nitrocellulose membranes using a wet-blot transfer system (Bio-Rad). To visualize the ZBP1-RNA complexes, the blots were exposed via autoradiography.

ZBP1 purification

Ten 15 cm diameter tissue culture dishes seeded with TLA HEK293T cells were each transfected with 5 μg pLenti6.3/TO/V5-DEST-ZBP1-3xFLAG using FuGENE 6 (Promega). 48 hours post-transfection cells were lysed in hypotonic buffer (10 mM Tris.HCl pH 7.4, 10 mM KCl, 1.5 mM $MgCl_2$) supplemented with protease inhibitor cocktail (Cell Signalling). Lysates were centrifuged at 100,000 g and supernatant was filtered through a 0.45 um PVDF-membrane and applied on a HiTrap heparin HP column (GE Healthcare) equilibrated in buffer B (20 mM Tris.HCl pH 7.4, 0.02% CHAPS and 0.5 mM DTT) and eluted with 0.5 M NaCl in buffer B. ZBP1-3xFLAG was then immunoprecipitated using anti-FLAG-coupled magnetic beads (Sigma Alrich, clone M2), washed 3 times in buffer C (50 mM Tris.HCl pH 7.4, 100 mM NaCl, 10% glycerol, 0.5% NP-40 (Igepal CA-630, Sigma), 0.5 mM EDTA, 0.5 mM EGTA). ZBP1-3xFLAG was eluted three times from the beads using 0.1 mg/ml 3xFLAG-peptide (Sigma Aldrich) in

50mM Tris.HCl pH 7.4 150mM NaCl 0.02% CHAPS. Finally, the eluate was concentrated using an Amicon Ultra-15 MWCO 10kDa tube.

Appendix Table S1: Primers for genotyping and cloning

Name	Sequence (5' to 3')	Purpose
gtZBP1Za1Za2-fwd	TGGCTTCAAATCTGAGCTTGGC	Genotyping Zbp1-Zα1Zα1 mutant mice
gtZBP1Za1Za2-rvs	CTATCTGGAGCTGAATGAAGGCA	
Zbp1-Exon2-fwd	TGACCACTGTAACCAATGCCACCT	PCR primers for sequencing of Exon 2 (Zα1)
Zbp1-Exon2-rvs	GACCTGAAACCTGACCTTCCCAAC	
Zbp1-Exon3-fwd	TGGTGTCGTCGTTCCTGTCTGCTT	PCR primers for sequencing of Exon 3 (Zα2)
Zbp1-Exon3-rvs	CTCGTGGCTCTTAATGTGCGTGTAG	
mZBP1fwd-Kozak	GCCGCCACCATGGCAGAAGCTCCTGTTG	TOPO-TA cloning in pCR8/GW/TOPO
mZBP1rvs-3xFLAG	CTTGTCATCGTCATCCTTGTAATCGATGTCATGATCTTTATAATCACCGTCATGGTCTTTGTAGTCTTGCTTGCTCAGTCCTGTG	
Mut-Zα1-fwd	CCCAAGAAAACCCTCGCTCAAGTCCTTGCCCGCCTGAAGAAGGAG	Overlap PCR for Zα1 mutation
Mut-Zα1-rvs	CTCCTTCTTCAGGCGGGCAAGGACTTGAGCGAGGGTTTTCTTGGG	
Mut-Zα2-fwd	ACAGCCAAAGAAGTGGCCCCACTCCTGGCTTCCATGAGAAATAAG	Overlap PCR for Zα2 mutation
Mut-Zα2-rvs	CTTATTTCTCATGGAAGCCAGGAGTGGGGCCACTTCTTTGGCTGT	
M36fwd-Kozak	AGGAGGACAGCTATGTATGAGCAAGAGGAACAAC	TOPO-TA cloning in pCR8/GW/TOPO
M36rvs	TCGATATCCCCGTGTCATC	

Appendix Table S2: Primers and probes for (RT)-qPCR

Gene	Assay Probe ID / Sequence (5' to 3')	Assay type
Ifi44	Mm00505670_m1 (Applied Biosystems)	Taqman
Ifit1	Mm00515153_m1 (Applied Biosystems)	Taqman
Oasl1	Mm00455081_m1 (Applied Biosystems)	Taqman
Ifnb	Mm00439552_s1 (Applied Biosystems)	Taqman
Gapdh	Cat. no. 4352932E (Applied Biosystems)	Taqman
gB-fwd	AGGGCTTGGAGAGGACCTACA	Taqman
gB-rvs	GCCCGTCGGCAGTCTAGTC	
gB-probe	5'FAM_AGCTAGACGACAGCCAACGCAACGA_3'TAMRA	
M36-fwd	GTGTGATGAAGGAAAGTACGTC	SYBR green
M36-rvs	CTGGAAGAAGGACACTAGACTG	
IE3-fwd	ATGTCGCCAACAAGATCCTC	SYBR green
IE3-rvs	ATATCTATGTTCATCTCGGGTCCT	

Appendix Table S3: Antibodies used for Western blot

Antigen	Supplier	Host	Cat. number	Dilution
Mouse ZBP1	A. Pichlmair	Rabbit polyclonal		1:2000
Mouse ZBP1	Adipogen (clone Zippy-1)	Mouse monoclonal	AG-20B-0010	1:2000
Mouse RIPK3	Novus Biologicals	Rabbit polyclonal	NBP1-77299	1:2000
Mouse MLKL	Millipore (clone 3H1)	Rat monoclonal	MABC604	1:1000
Mouse MLKL	Sigma Aldrich (for trimer and oligomers)	Rabbit polyclonal	SAB1302339	1:2000
Mouse P-S345 MLKL	Abcam (EPR9515(2))	Rabbit monoclonal	ab196436	1:2000
Mouse ISG15	A. Pichlmair	Rabbit polyclonal		1:2000
MCMV IE1	Q. Tang	Mouse monoclonal		1:1000
HA-tag	sc-7392 (clone F-7)	Mouse monoclonal	sc-7392	1:5000
FLAG-tag	Sigma Aldrich (clone M2)	Mouse monoclonal (HRP-coupled)	A8592	1:20,000
V5-tag	Life Technologies	Mouse monoclonal (HRP-coupled)	R96125	1:2000
Beta-actin	Sigma Aldrich	Mouse monoclonal (HRP-coupled)	A3854	1:500,000

Appendix Table S4: siRNA sequences

siRNA	Sequence (5’ to 3’) / cat. No.
IE3-1-sense	CUAAGAAGCAUAAGAACAAUU
IE3-1-antisense	UUGUUCUUAUGCUUCUUAGUU
IE3-2-sense	GAAGAUAAGUCCAGGAAGUUU
IE3-2-antisense	5'P_ACUUCCUGGACUUAUCUUCUU
IE3-3-sense	GGAUGAAGAUGAUGAGGAUUU
IE3-3-antisense	AUCCUCAUCAUCUUCAUCCUU
IE3-4-sense	GUGAGUAGUGGGAGUGAUAUU
IE3-4-antisense	UAUCACUCCCACUACUCACUU
Control siRNA	ON-TARGETplus Non-targeting Control Pool
