# Supplementary material for: Sensing of viral and endogenous RNA by ZBP1/DAI induces necroptosis
Source: EMBO J. 2017 Jul 17;36(17):2529–43. doi: 10.15252/embj.201796476 (PMC5579359; doi:10.15252/embj.201796476)
Supplement: Supplementary file 2 — Expanded View Figures PDF [file EMBJ-36-2529-s002.pdf]

Expanded View Figures

Figure EV1. ZBP1-dependent necroptosis requires intact ZBP1 ZBDs (related to Fig 1).

A, B SV40 large T antigen-immortalised MEFs of the indicated genotypes were treated or not with 100 U/ml of IFN-A/D for 16 h. (A) Cell extracts were subjected to Western blot analysis using the indicated antibodies. Asterisk (*) indicates non-specific bands. (B) Cells were treated with 30 ng/ml TNF and 20 μM zVAD (TZ) or were infected with the indicated viruses at an MOI of 10. After 16 h, cell viability was assessed using CellTiter-Glo reagent. Values for untreated cells were set to 100%.

C HEK293T cells were transfected with 50 ng NF-κB firefly luciferase and 25 ng *Renilla* luciferase reporter plasmids, together with an expression vector for RIPK3. Luciferase activity was measured after 24 h, and the ratio of firefly and *Renilla* luciferase was set to 1 for control cells that did not receive RIPK3 plasmid.

D NIH3T3 cells were treated with IFN-A/D for 16 h, and cell extracts were analysed by Western blot (top). Asterisk (*) indicates a non-specific band. ZBP1-3xFLAG-reconstituted NIH3T3 cells were also tested by Western blot (bottom).

E ZBP1-reconstituted NIH3T3 cells were infected as indicated and analysed as in (B).

F Cells were treated with 1,000 U/ml of IFN-A/D for 16 h, and cell extracts were analysed by Western blot. Arrows indicate endogenous (lower band) and exogenous 3xFLAG-tagged ZBP1 (upper band).

G Cells were infected with MCMV-M45^{mutRHIM} at an MOI of 10 or treated with TZ and analysed as in (B).

H Cell death was monitored upon infection or TZ treatment using an in-incubator imaging platform (Incucyte) and the dye YOYO-3, which stains dead cells.

Data information: Data are representative of three or more independent experiments. Panels (B, C, E, G and H) represent mean ± SD (n = 3). *$P < 0.05$, **$P < 0.01$, ***$P < 0.001$; two-way ANOVA.
Source data are available online for this figure.

 © 2017 The Authors

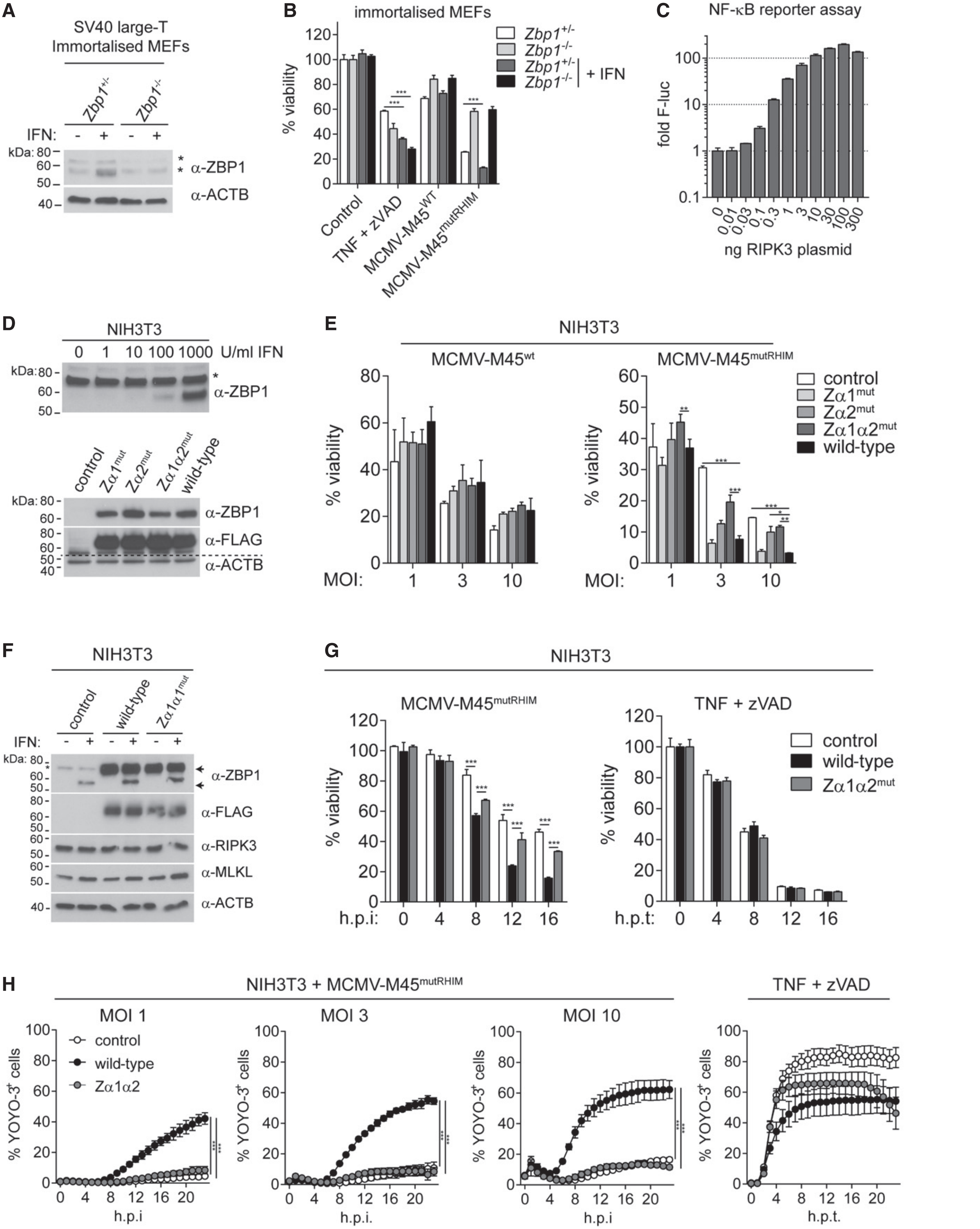
A
SV40 large-T
Immortalised MEFs
Zbp1+/-
Zbp1-/-
IFN:
kDa: 80
60
50
40
α-ZBP1
α-ACTB
B
immortalised MEFs
% viability
Zbp1+/-
Zbp1-/-
Zbp1+/-
Zbp1-/-
+ IFN
Control
TNF + zVAD
MCMV-M45WT
MCMV-M45mutRHIM
C
NF-κB reporter assay
fold F-luc
ng RIPK3 plasmid
D
NIH3T3
U/ml IFN
α-ZBP1
control
Zα1mut
Zα2mut
Zα1α2mut
wild-type
α-FLAG
α-ACTB
E
NIH3T3
MCMV-M45wt
MCMV-M45mutRHIM
% viability
control
Zα1mut
Zα2mut
Zα1α2mut
wild-type
MOI:
F
NIH3T3
control
wild-type
Zα1α1mut
IFN:
α-ZBP1
α-FLAG
α-RIPK3
α-MLKL
α-ACTB
G
NIH3T3
MCMV-M45mutRHIM
TNF + zVAD
% viability
control
wild-type
Zα1α2mut
h.p.i:
h.p.t:
H
NIH3T3 + MCMV-M45mutRHIM
TNF + zVAD
MOI 1
MOI 3
MOI 10
% YOYO-3+ cells
control
wild-type
Zα1α2
h.p.i
h.p.i.
h.p.t.

Figure EV1.

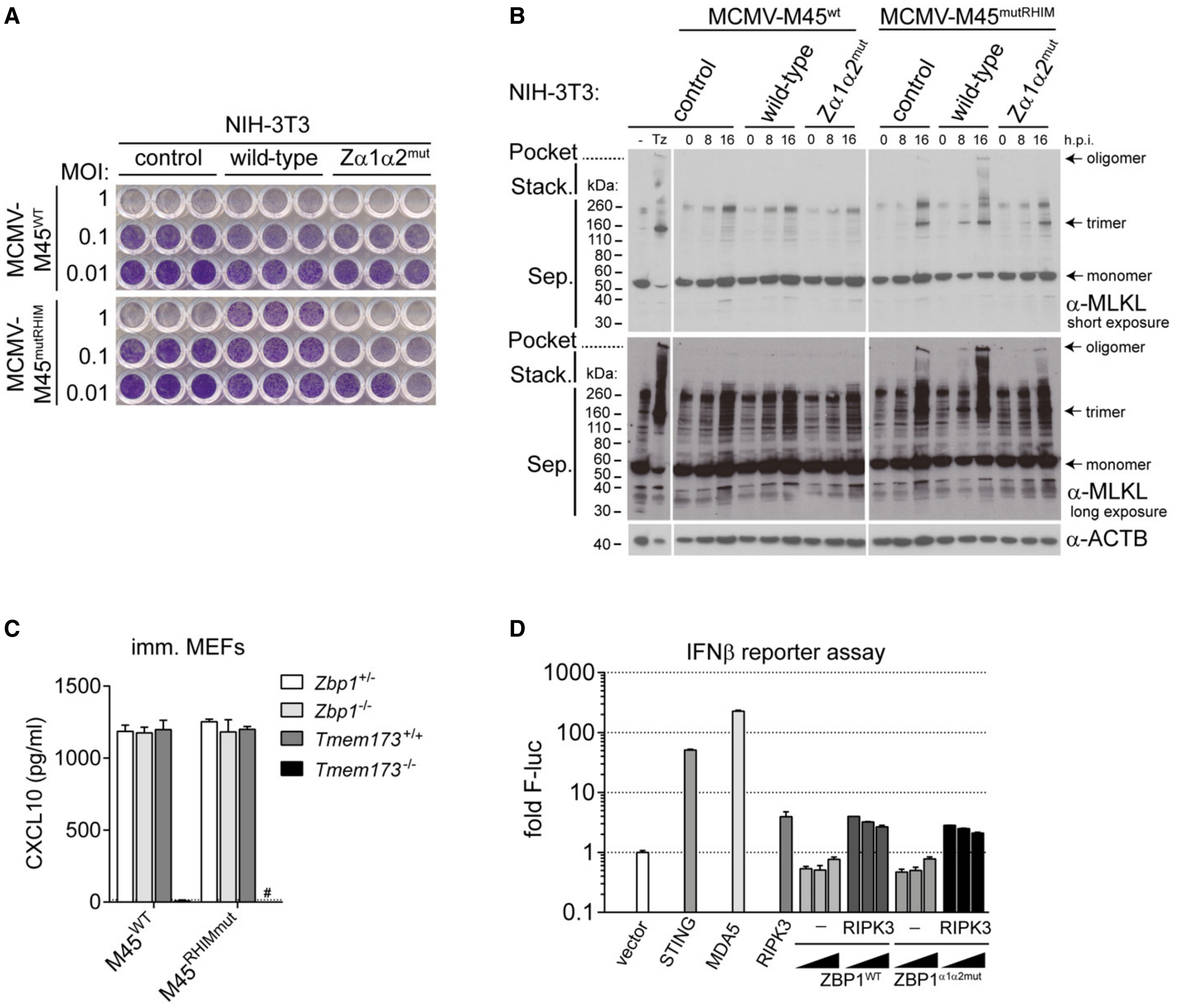

Figure EV2. ZBP1 controls MCMV replication in NIH3T3 cells (related to Fig 2).

A NIH3T3 cells reconstituted with wild-type or mutant ZBP1 were infected as indicated. After 5 days, cells were fixed and stained with crystal violet.

B NIH3T3 cells reconstituted with wild-type or mutant ZBP1 were infected as indicated using an MOI of 3. Control cells were treated with TZ. After 0, 8 or 16 h, cell lysates were subjected to Western blot analysis under non-reducing conditions using the indicated antibodies. Short and long exposures of α-MLKL Western blot are shown.

C CXCL10 was analysed by ELISA in supernatants from MEFs of the indicated genotypes infected with MCMV-M45^{WT} or MCMV-M45^{mutRHIM} (MOI = 3; 8 h). $^{\#}$not detected. The dotted line represents the lower limit of detection.

D HEK293T cells were transfected with 125 ng IFNβ firefly luciferase and 25 ng *Renilla* luciferase reporter plasmids, together with expression vectors for HA-STING (500 ng), MDA5 (500 ng), RIPK3 (50 ng) or ZBP1-3xFLAG (20, 100, 500 ng). Luciferase activity was measured after 24 h and the ratio of firefly and *Renilla* luciferase was set to 1 for control cells transfected with empty vector.

Data information: Data are representative of two or more independent experiments. Panels (C and D) show mean ± SD (n = 3).
Source data are available online for this figure.

 © 2017 The Authors

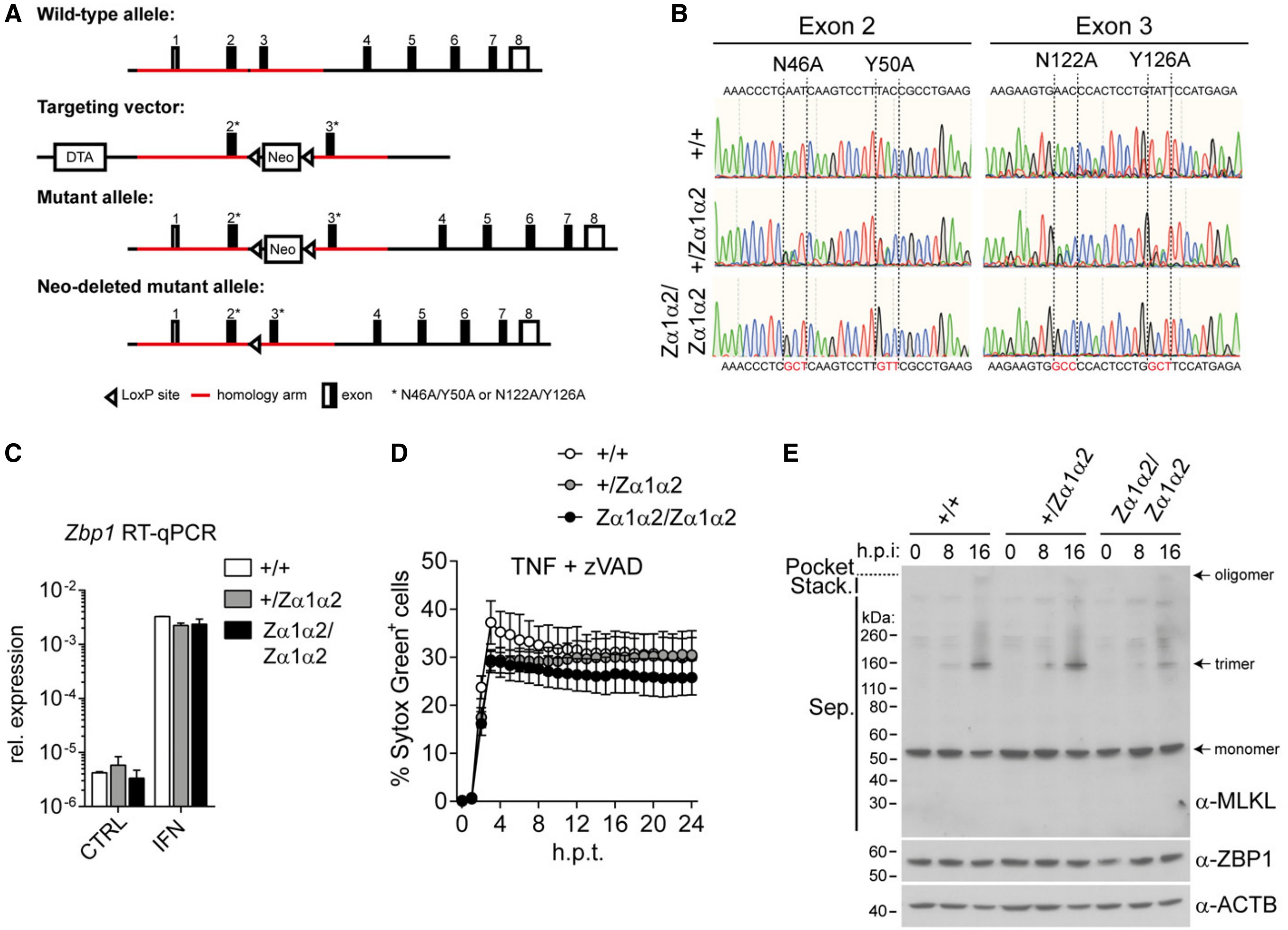

Figure EV3. Validation of ZBP1-Zα1α2^{mut} knock-in (related to Fig 3).

A Targeting strategy. See Materials and Methods for further details.
B DNA fragments encompassing exon 2 or 3 were PCR-amplified from genomic DNA from primary MEFs of the indicated genotypes and were then sequenced.
C Primary MEFs of the indicated genotypes were treated with 100 U/ml IFN-A/D for 16 h. RNA was extracted, and *Zbp1* expression was analysed by RT qPCR.
D Cell death was monitored upon treatment with TZ using an in-incubator imaging platform (Incucyte) and the dye Sytox Green that stains dead cells.
E Non-reducing SDS–PAGE and Western blot for MLKL from samples from Fig 3D.

Data information: Data are representative of two or more independent experiments. Panels (C and D) show mean ± SD (n = 3).
Source data are available online for this figure.

Figure EV4. ZBP1 binds newly synthesised RNA in MCMV-infected cells (related to Fig 4).

A NIH3T3 cells (top panel) or immortalised *Zbp1*$^{-/-}$ MEFs (lower panel) were treated with indicated concentrations of ganciclovir (GCV) and infected with MCMV-M45^{mutRHIM} or UV-C inactivated MCMV-M45^{mutRHIM} as indicated. After 5 days, cells were fixed and stained with crystal violet.
B RNA samples from the experiment shown in Fig 4B and C were analysed by RT qPCR.
C Structure of the MCMV IE1/3 gene. Four different siRNAs specifically targeting IE3 (exon 5) were designed.
D NIH3T3 cells were transfected with the indicated siRNAs and were then infected with MCMV-IE1/3-GFP. GFP-expressing cells were enumerated using Incucyte analysis.
E NIH3T3 cells expressing wild-type or mutant ZBP1 were infected with MCMV-M45^{mutRHIM} (MOI 3) and 3 h post-infection 30 ng/ml TNF was added to the cells. Cell death was analysed as in Fig 4F.
F NIH3T3 cells expressing wild-type ZBP1 were transfected with the indicated siRNAs, infected with MCMV-M45^{mutRHIM} (MOI 3) and 3 h post-infection 30 ng/ml TNF was added to the cells. Cell death was analysed as in Fig 4F.
G, H Immortalised *Zbp1*$^{-/-}$ MEFs reconstituted with wild-type ZBP1 were infected with MCMV-M45^{mutRHIM} (MOI = 10) in the presence of neutralising anti-IFNAR1 or isotype control antibodies. (G) 16 h post-infection viability was assessed as in Fig 1B. (H) RNA samples were collected 8 h after infection and analysed by RT–qPCR.

Data information: Data are representative of two or more independent experiments. Panels (B, D–H) show mean ± SD (n = 3). *P < 0.05; one-way ANOVA.

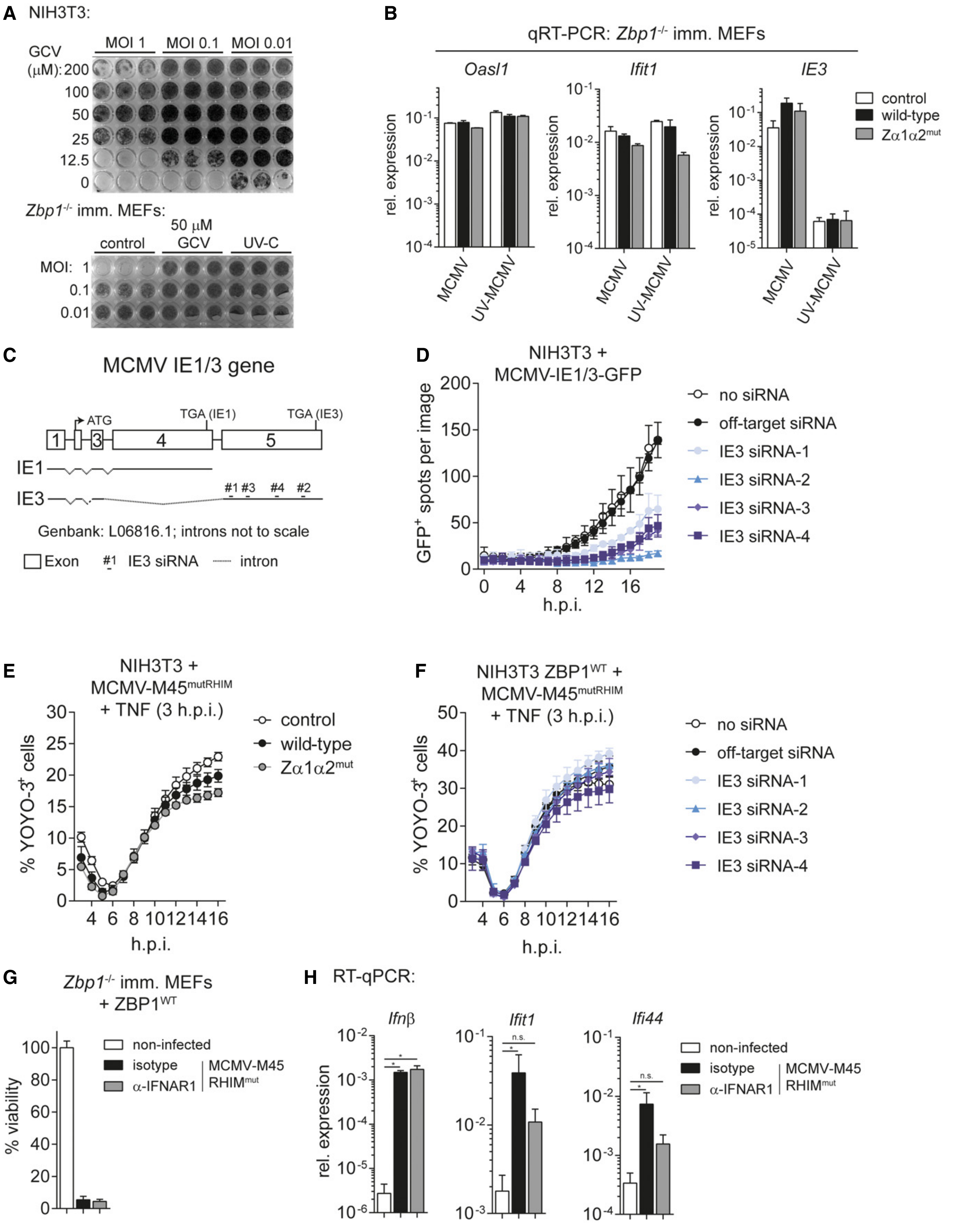

Figure EV4.

© 2017 The Authors

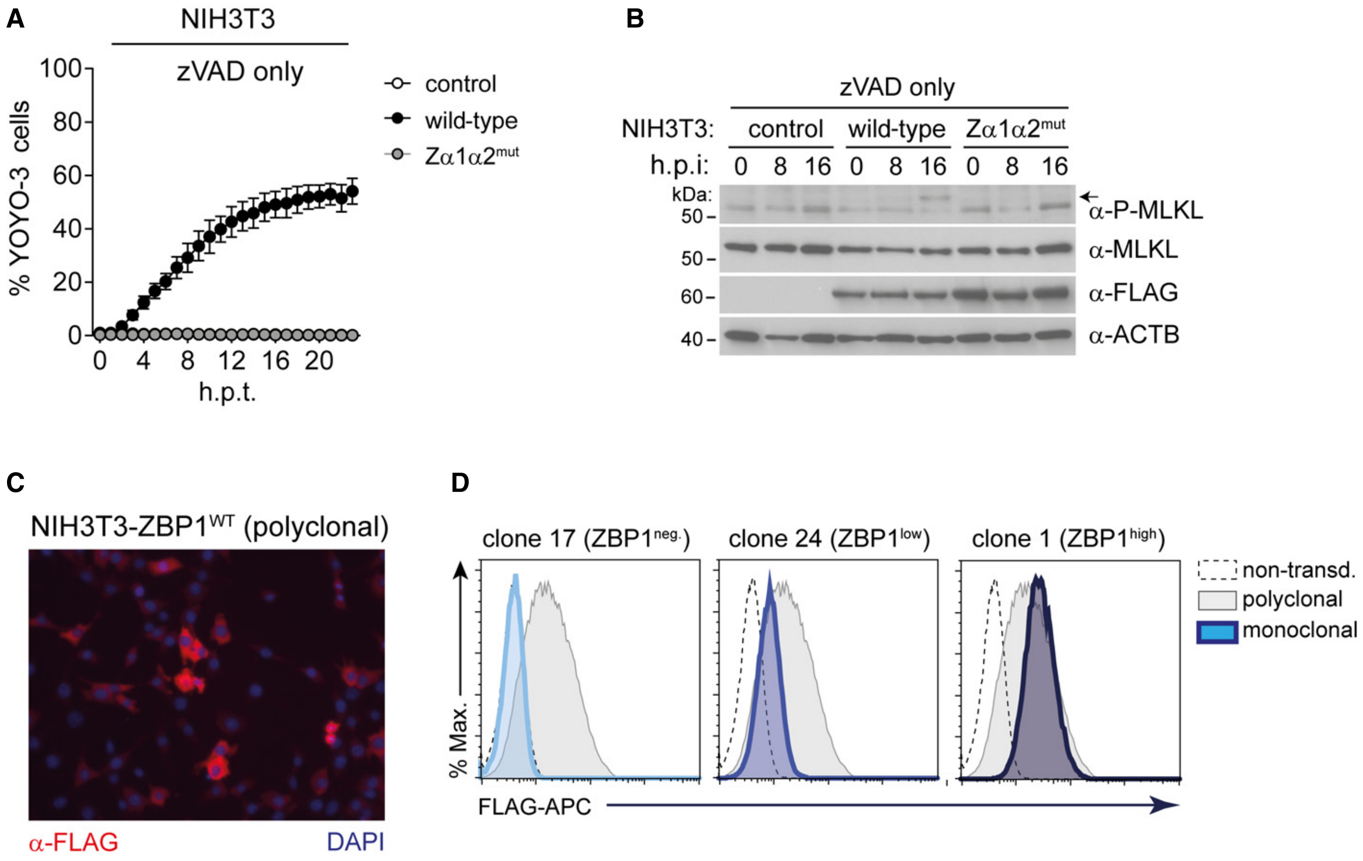

Figure EV5. Generation of monoclonal ZBP1-expressing cell lines (related to Fig 5).

A NIH3T3 cells were treated with zVAD as in Fig 5A and viability was measured using an in-incubator imaging platform (Incucyte) and the dye YOYO-3 that stains dead cells.

B NIH3T3 cells were treated with 20 μM zVAD for 0, 8 and 16 h, and cell lysates were analysed by Western blot. The arrow indicates phosphorylated MLKL.

C Immunofluorescence analysis of polyclonal wild-type ZBP1-3xFLAG-expressing NIH3T3 cells. Cells were stained with α-FLAG-HRP and secondary anti-HRP-Alexa Fluor 594 antibodies.

D Clonal cell lines were derived by limiting dilution and were then analysed by flow cytometry using α-FLAG-APC antibody. Selected ZBP1 negative, low and high clones are shown.

Data information: Panel (A) shows mean ± SD (n = 3). Data are representative of two independent experiments.
Source data are available online for this figure.

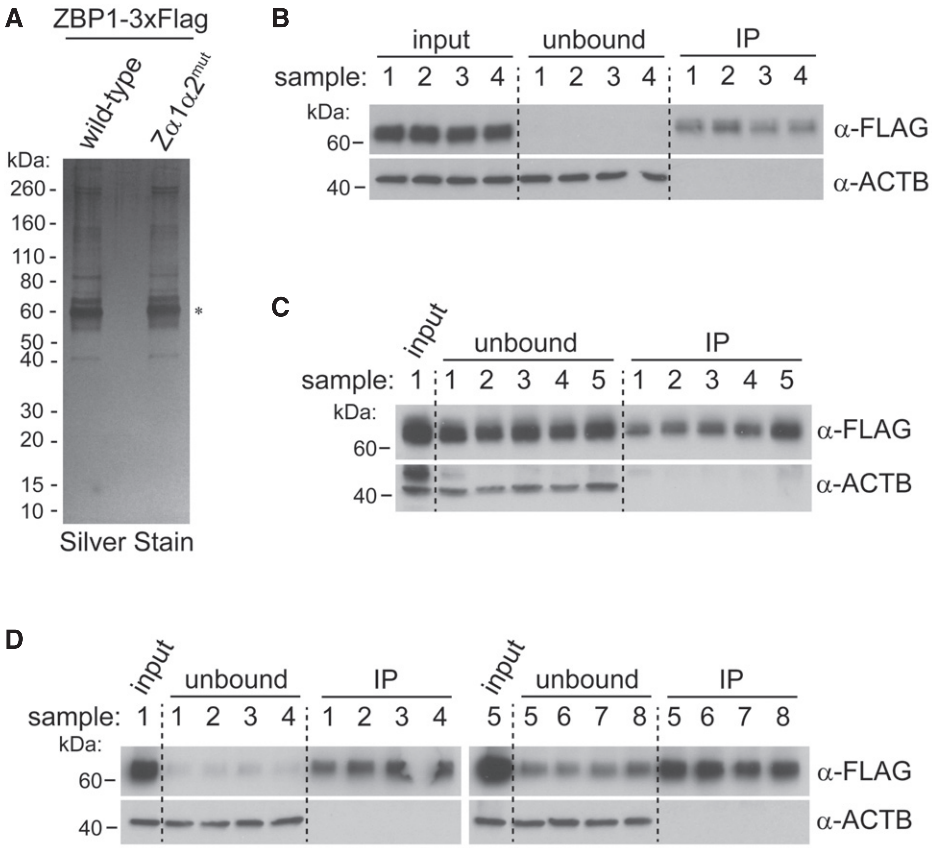

Figure EV6. Purification of recombinant ZBP1 and Western blots for CLIP experiments (related to Fig 6).

A HEK293T cells were transfected with plasmids expressing wild-type or mutant ZBP1-3xFLAG. Tagged ZBP1 was then purified by α-FLAG immunoprecipitation and analysed by SDS–PAGE and silver staining. Asterisk (*) indicates silver stain of recombinant ZBP1.

B–D Western blots for input, unbound and immunoprecipitated samples corresponding to the autoradiograms shown in Fig 6B–D.

Data information: Data are representative of three independent experiments.

Source data are available online for this figure.

 © 2017 The Authors
