# Supplementary material for: Sensing of viral and endogenous RNA by ZBP1/DAI induces necroptosis
Source: EMBO J. 2017 Jul 17;36(17):2529–43. doi: 10.15252/embj.201796476 (PMC5579359; doi:10.15252/embj.201796476)
Supplement: Supplementary file 3 — Source Data for Expanded View [file EMBJ-36-2529-s009.zip › Sourcedata_EV1/SourcedataEV1.pdf]

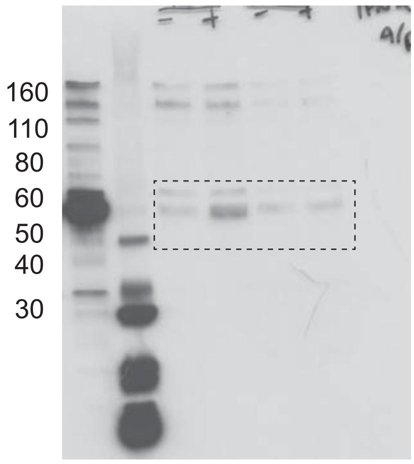

Figure EV1A_ZBP1

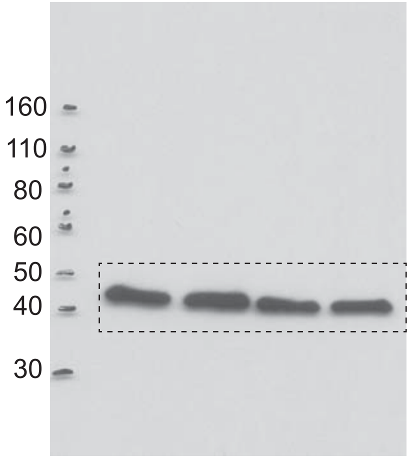

Figure EV1A_ACTB

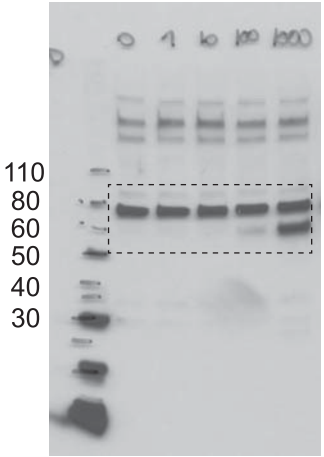

Figure EV1D_ZBP1

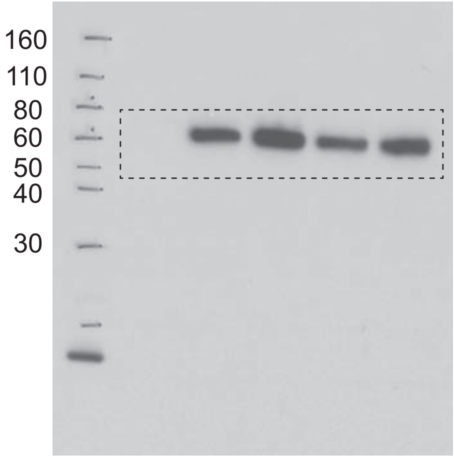

Figure EV1D_ZBP1 (lower panel)

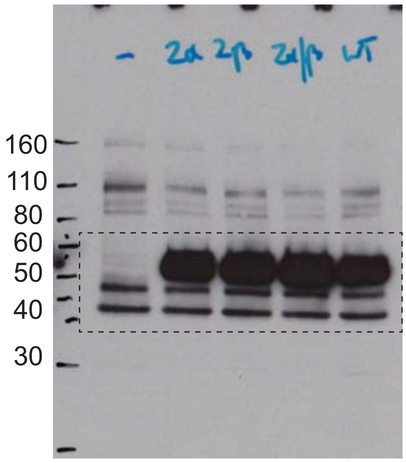

Figure EV1D_FLAG/ACTB (lower panel)

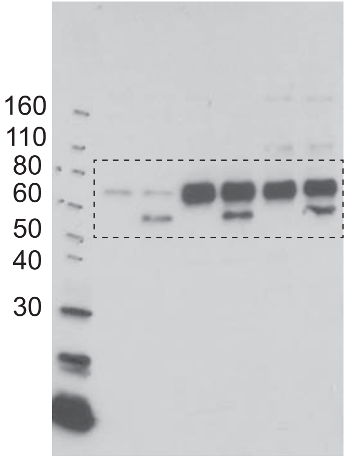

Figure EV1F_ZBP1

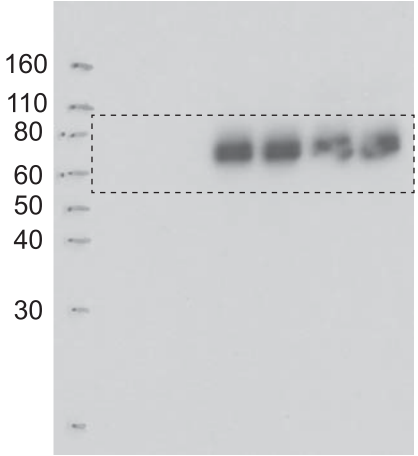

Figure EV1F_FLAG

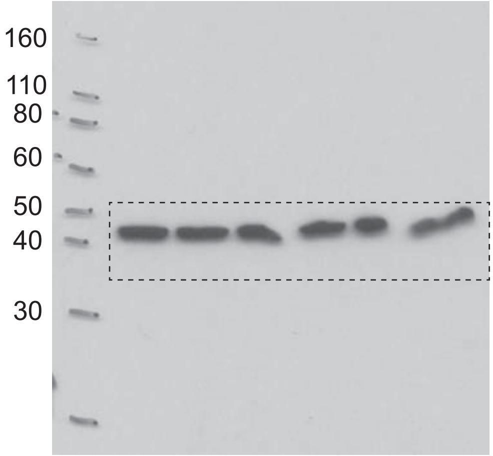

Figure EV1F_RIPK3

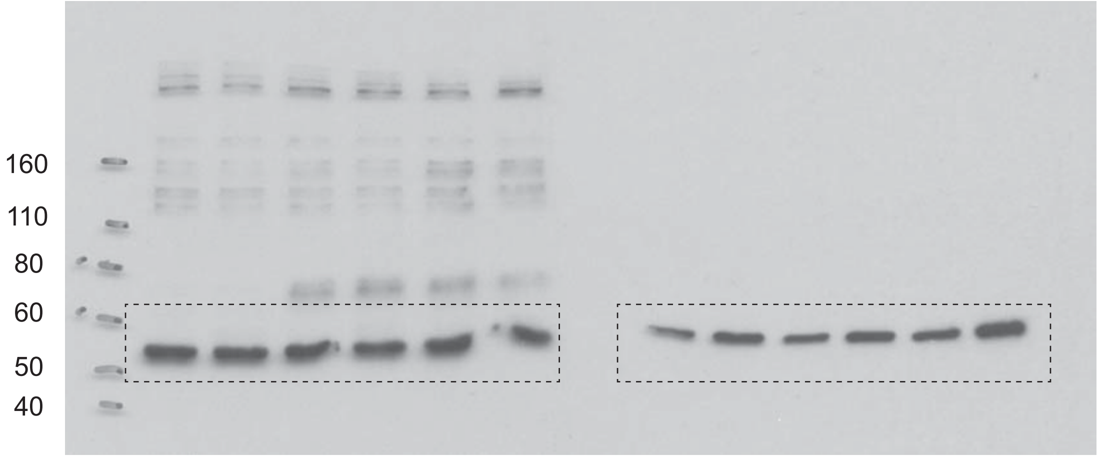

Figure EV1F_RIPK3

Figure EV1F_MLKL
