# Supplementary material for: Sensing of viral and endogenous RNA by ZBP1/DAI induces necroptosis
Source: EMBO J. 2017 Jul 17;36(17):2529–43. doi: 10.15252/embj.201796476 (PMC5579359; doi:10.15252/embj.201796476)
Supplement: Supplementary file 3 — Source Data for Expanded View [file EMBJ-36-2529-s009.zip › Sourcedata_EV6/SourcedataEV6.pdf]

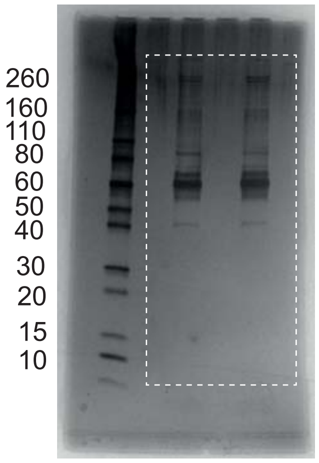

Figure EV6A_Silver Stain

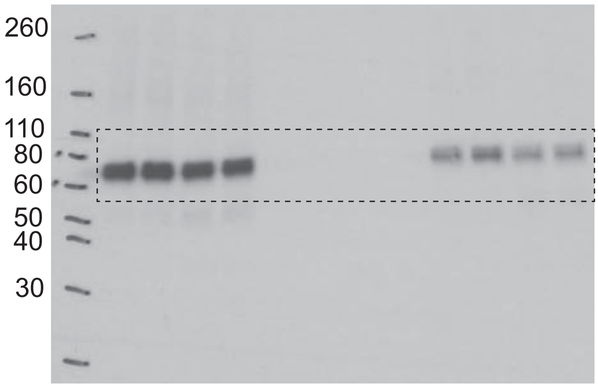

Figure EV6B_FLAG

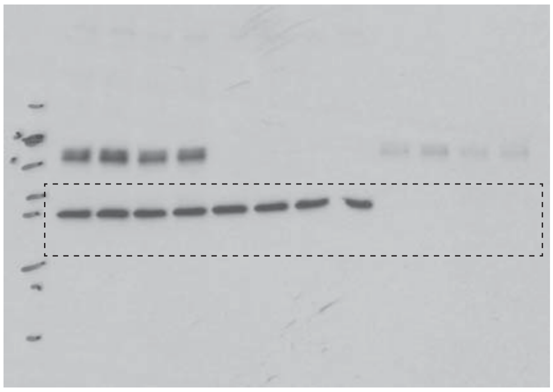
Figure EV6B_ACTB

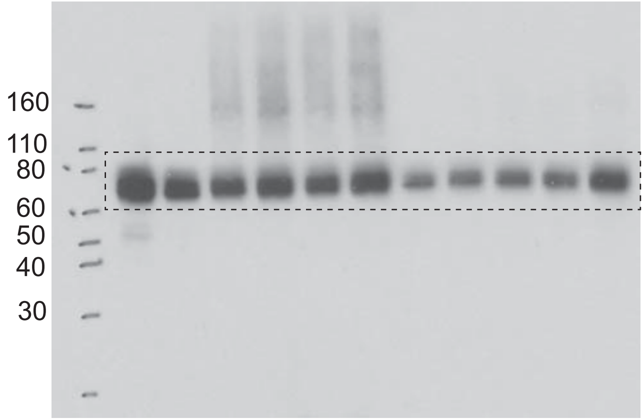

Figure EV6C_FLAG

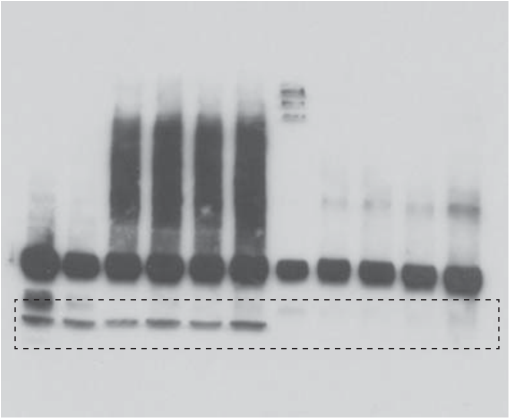
Figure EV6C_ACTB

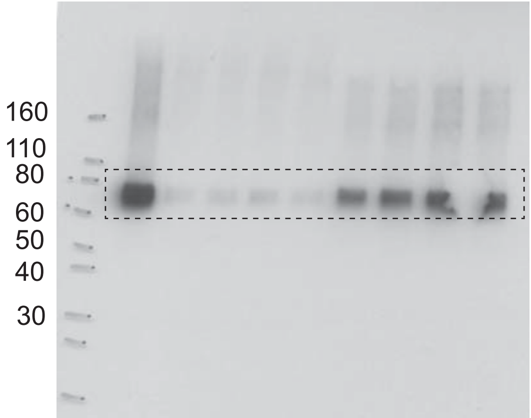

Figure EV6D_FLAG (WT)

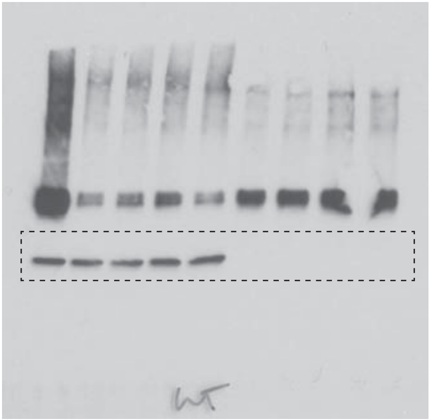
Figure EV6D_ACTB (WT)

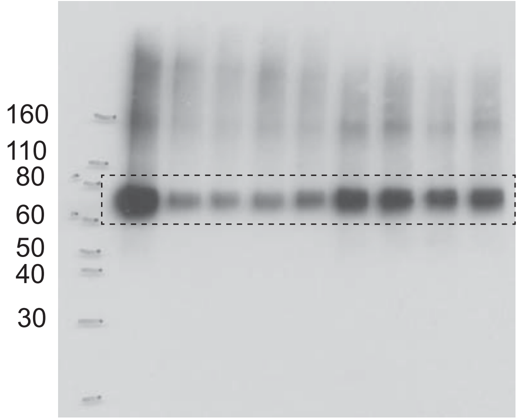

Figure EV6D_FLAG (mutant)

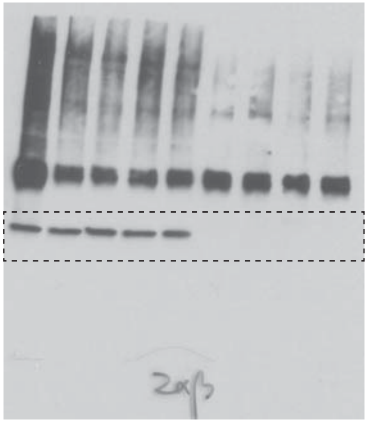
Figure EV6D_ACTB (mutant)
