# Supplementary figures and images for: Sensing of viral and endogenous RNA by ZBP1/DAI induces necroptosis
Source: EMBO J. 2017 Jul 17;36(17):2529–43. doi: 10.15252/embj.201796476 (PMC5579359; doi:10.15252/embj.201796476)

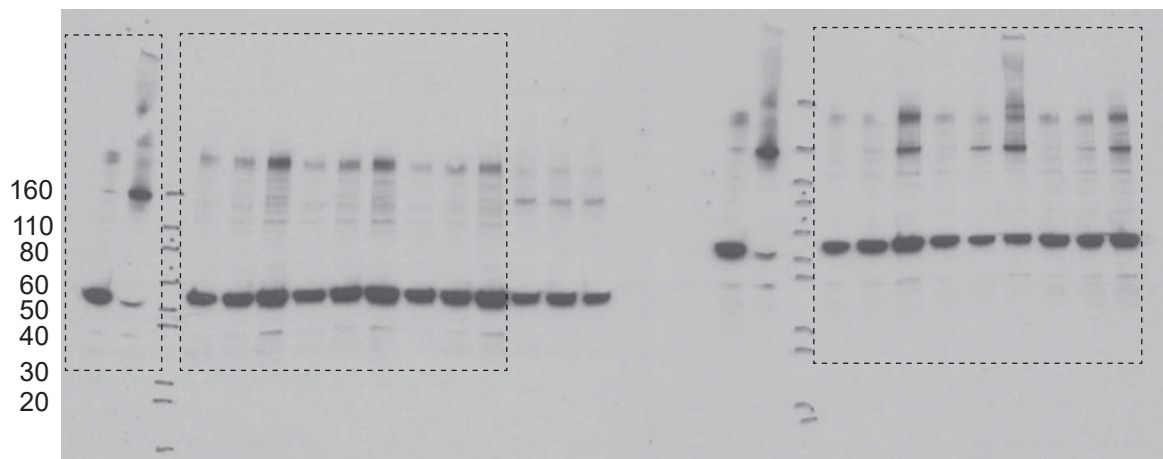

Figure EV2B\_MLKL (short exposure)

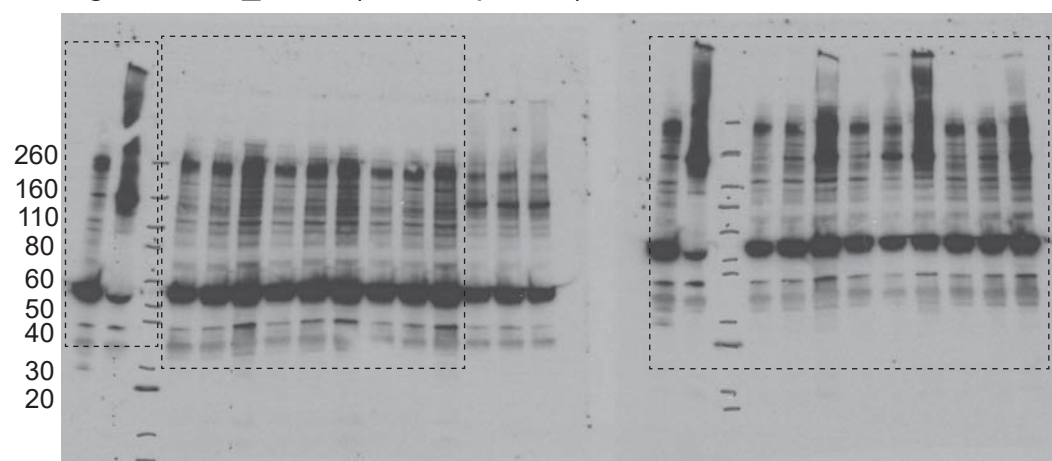

Figure EV2B\_MLKL (long exposure)

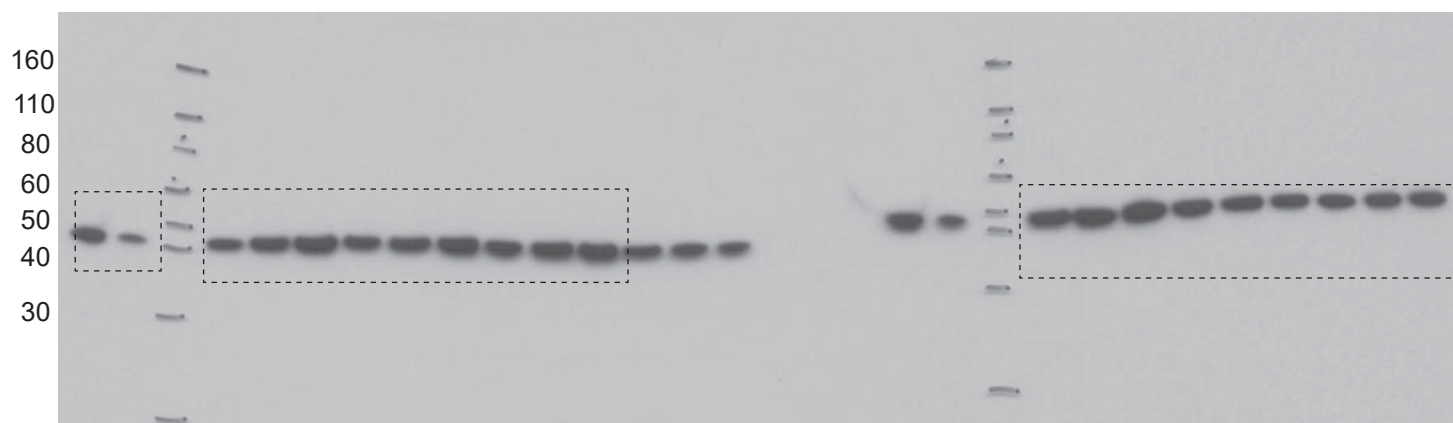

Figure EV2B\_ACTB

Supplement: Supplementary file 3 — Source Data for Expanded View [file EMBJ-36-2529-s009.zip › Sourcedata_EV2/SourcedataEV2.pdf]

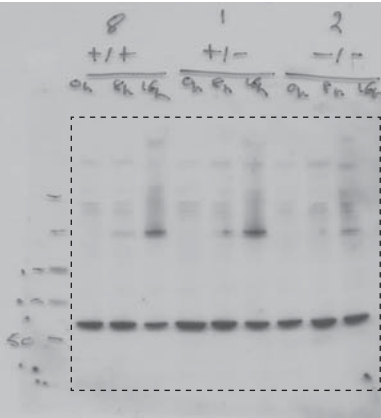

Figure EV3\_MLKL

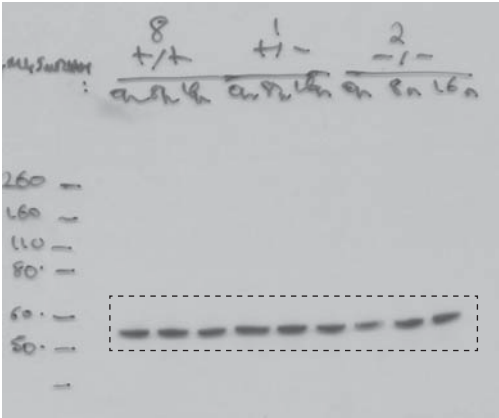

Figure EV3\_ZBP1

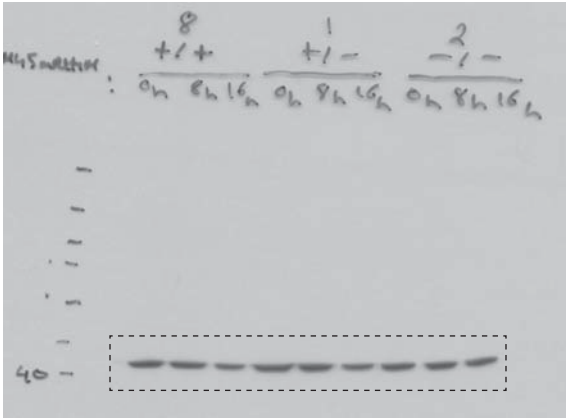

Figure EV3\_ACTB

Supplement: Supplementary file 3 — Source Data for Expanded View [file EMBJ-36-2529-s009.zip › Sourcedata_EV3/SourcedataEV3.pdf]

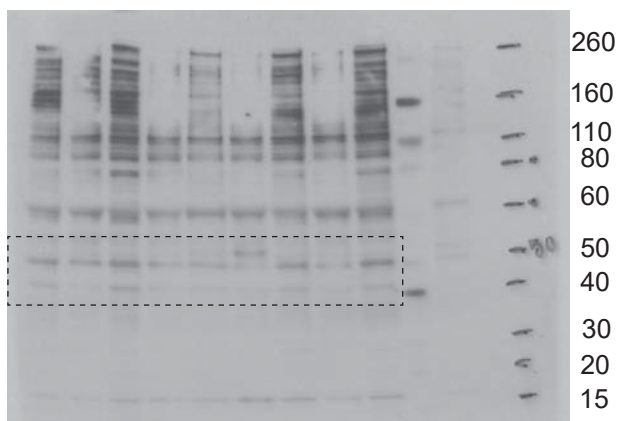

Figure EV5\_P-MLKL

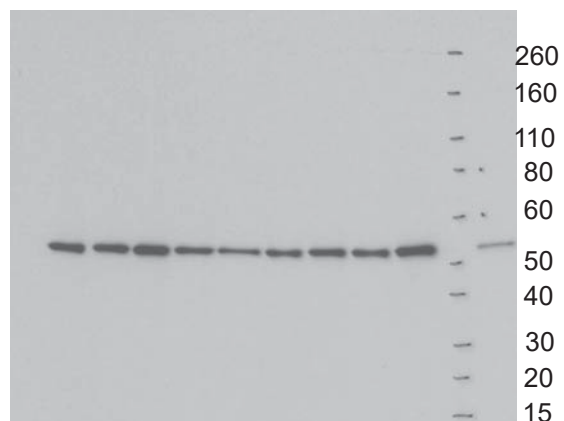

Figure EV5\_MLKL

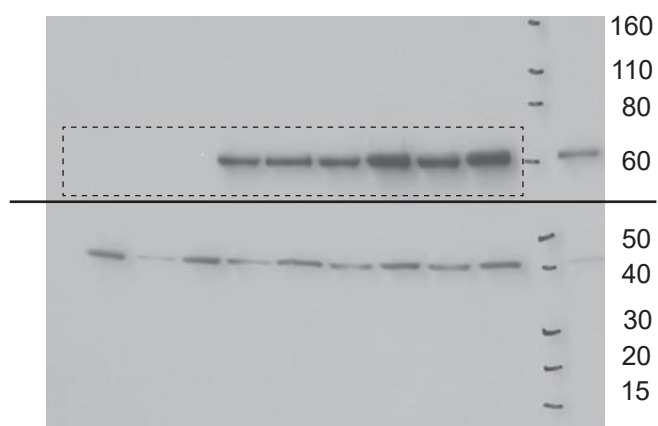

Figure EV5\_FLAG

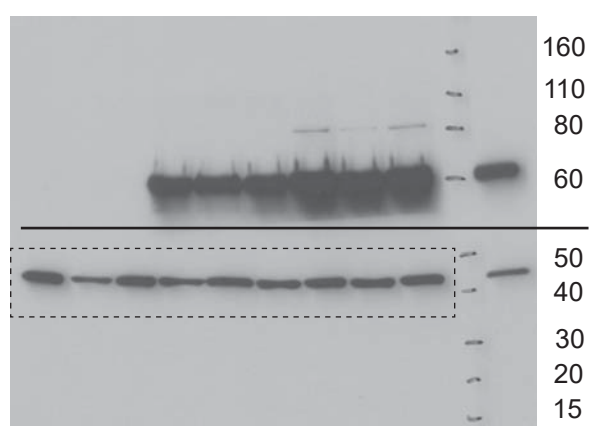

Figure EV5\_ACTB

Supplement: Supplementary file 3 — Source Data for Expanded View [file EMBJ-36-2529-s009.zip › Sourcedata_EV5/SourcedataEV5.pdf]

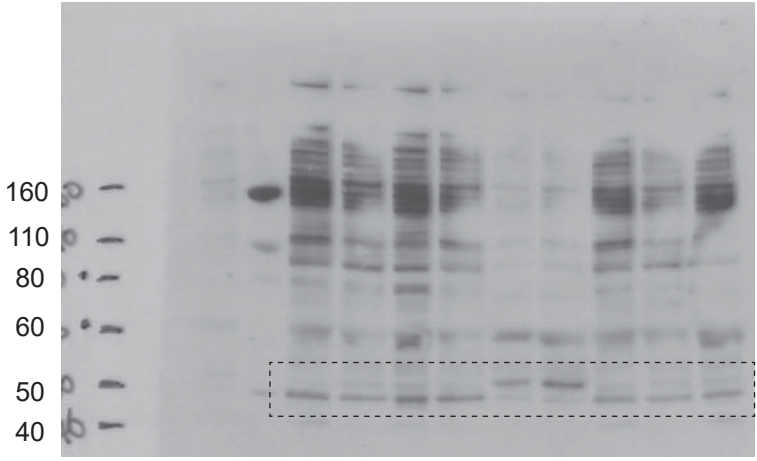

Figure 2C\_P-MLKL

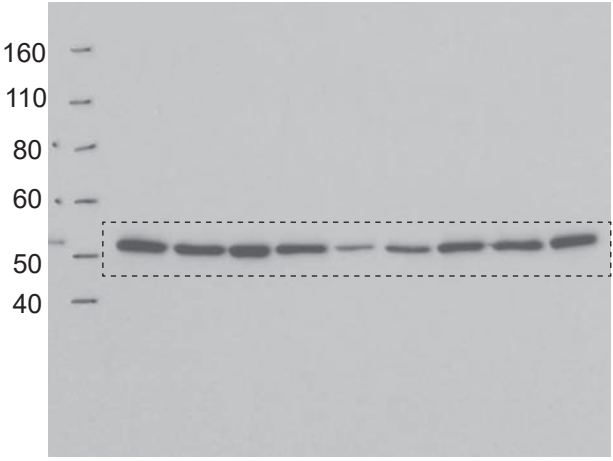

Figure 2C\_MLKL

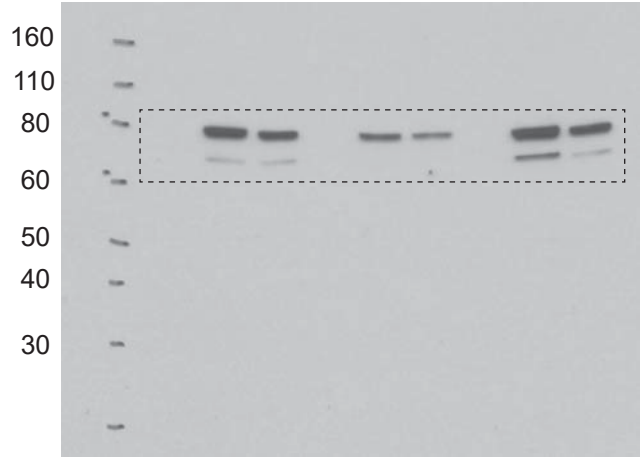

Figure 2C\_IE1

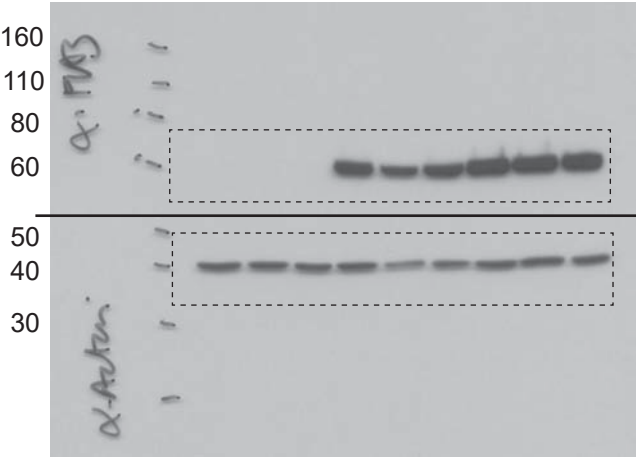

Figure 2C\_FLAG/ACTB

Supplement: Supplementary file 6 — Source Data for Figure 2 [file EMBJ-36-2529-s004.pdf]

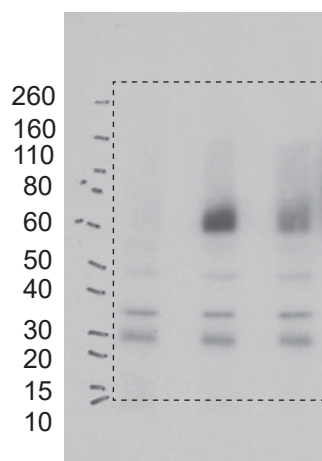

Figure 4G\_autoradiogram

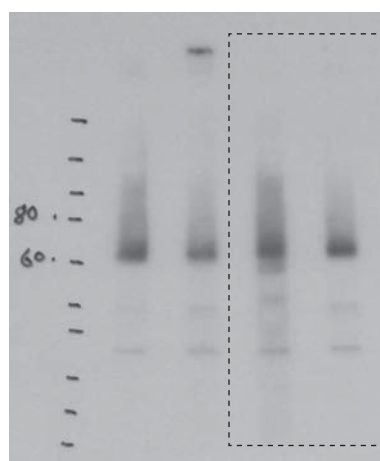

Figure 4H\_autoradiogram

Supplement: Supplementary file 8 — Source Data for Figure 4 [file EMBJ-36-2529-s006.pdf]

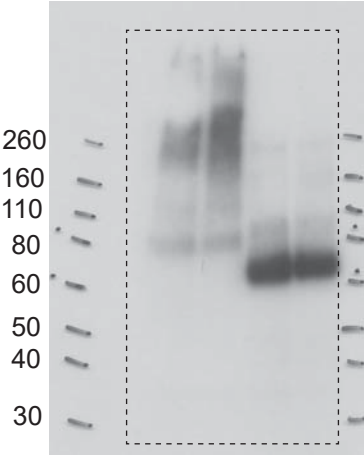

Figure 6B\_ autoradiogram

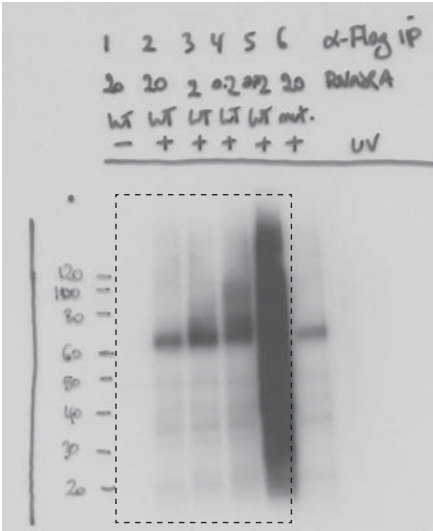

Figure 6C\_ autoradiogram

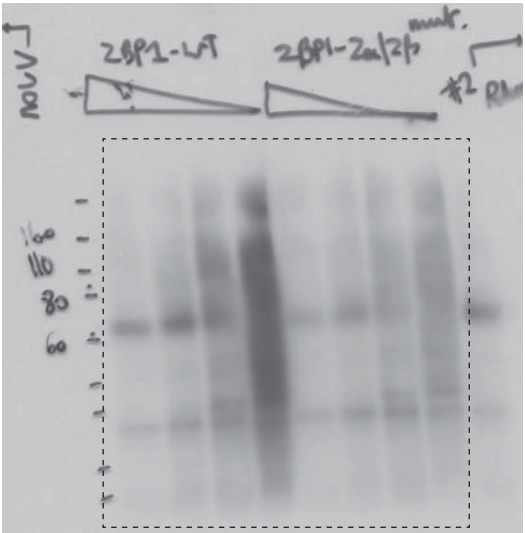

Figure 6D\_ autoradiogram

Supplement: Supplementary file 10 — Source Data for Figure 6 [file EMBJ-36-2529-s008.pdf]
