# Supplementary material for: Sensing of viral and endogenous RNA by ZBP1/DAI induces necroptosis
Source: EMBO J. 2017 Jul 17;36(17):2529–43. doi: 10.15252/embj.201796476 (PMC5579359; doi:10.15252/embj.201796476)
Supplement: Supplementary file 5 — Source Data for Figure 1 [file EMBJ-36-2529-s003.pdf]

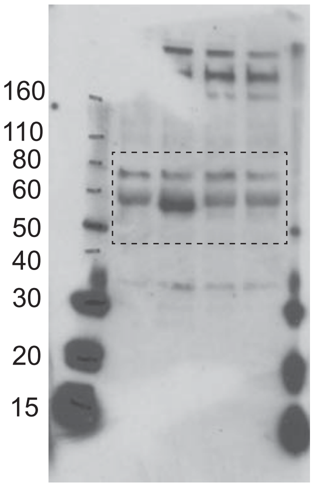

Figure 1A - ZBP1

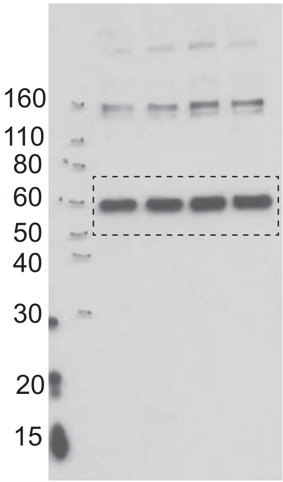

Figure 1A - RIPK3

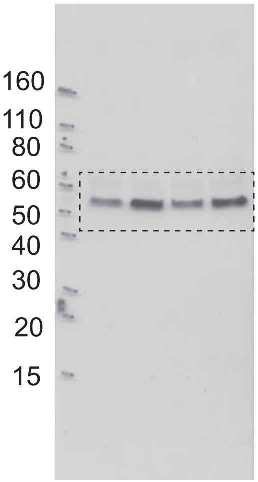

Figure 1A - MLKL

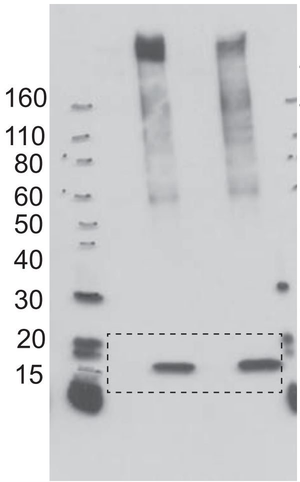

Figure 1A - ISG15

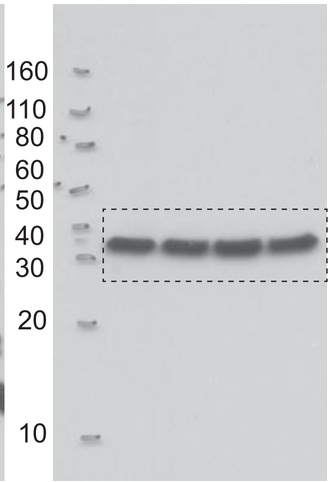

Figure 1A - ACTB

(top = +RIPK3; bottom = w/o RIPK3)

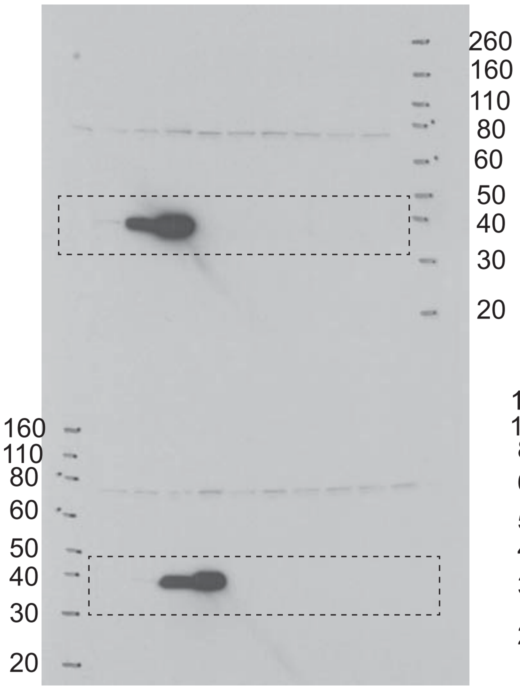

Figure 1E - HA

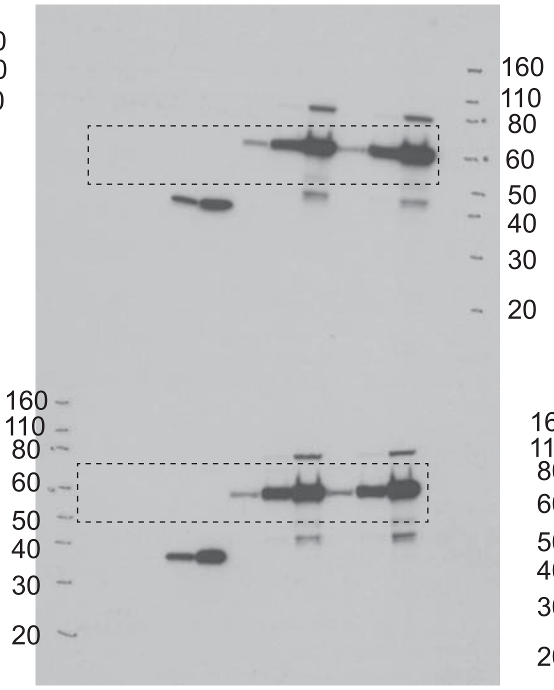

Figure 1E - FLAG

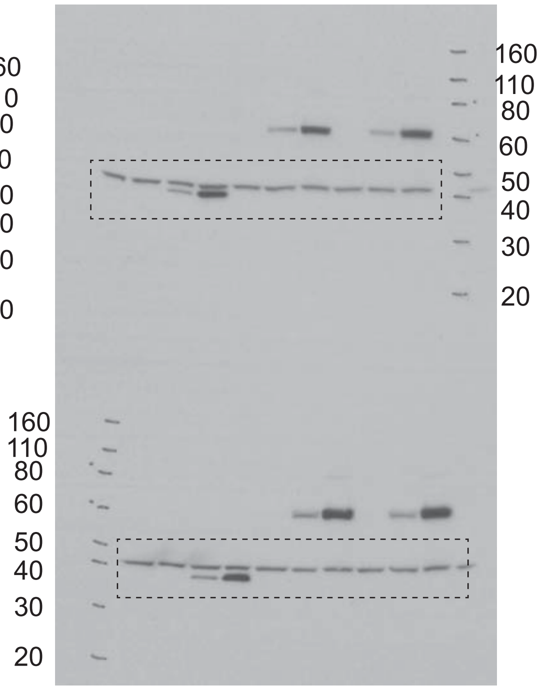

Figure 1E - ACTB

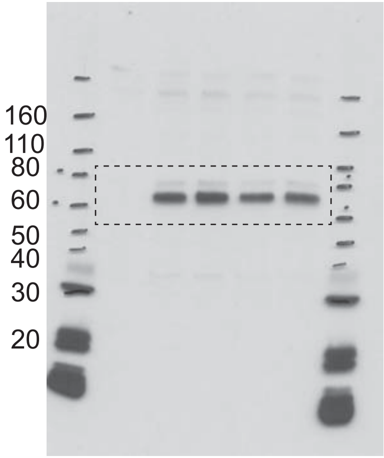

Figure 1F - ZBP1

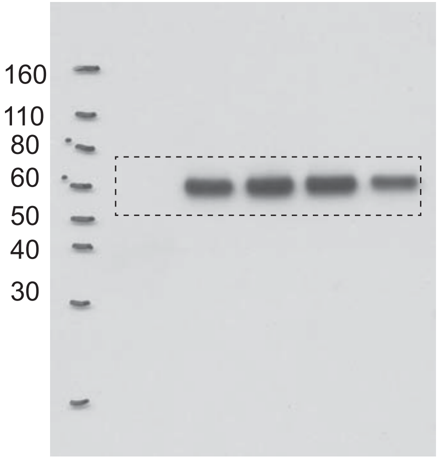

Figure 1F - FLAG

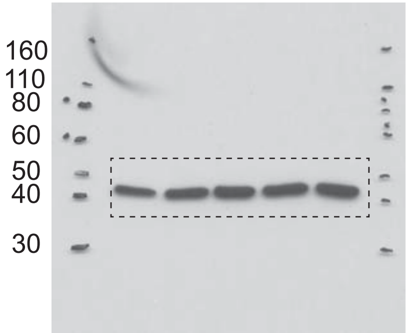

Figure 1F - ACTB
