# Supplementary material for: Sensing of viral and endogenous RNA by ZBP1/DAI induces necroptosis
Source: EMBO J. 2017 Jul 17;36(17):2529–43. doi: 10.15252/embj.201796476 (PMC5579359; doi:10.15252/embj.201796476)
Supplement: Supplementary file 7 — Source Data for Figure 3 [file EMBJ-36-2529-s005.pdf]

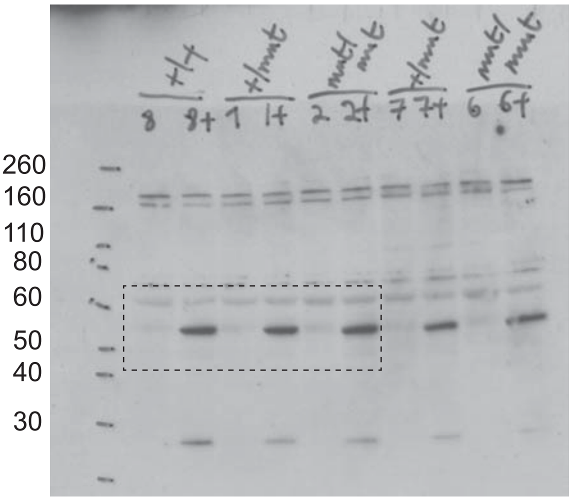

Figure 3A_ZBP1 (Zippy-1)

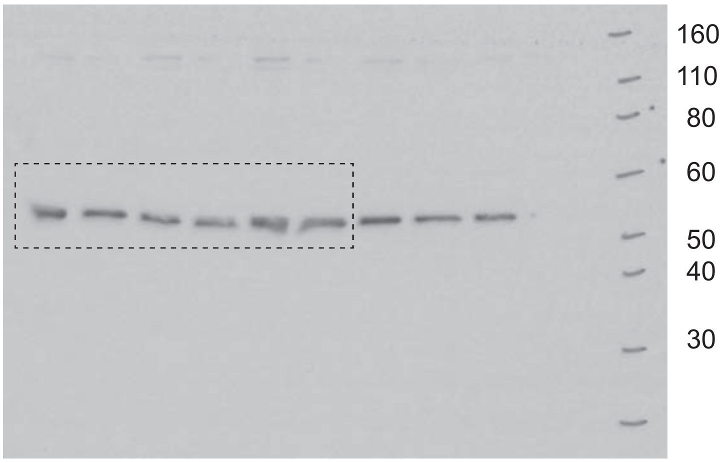

Figure 3A_RIPK3

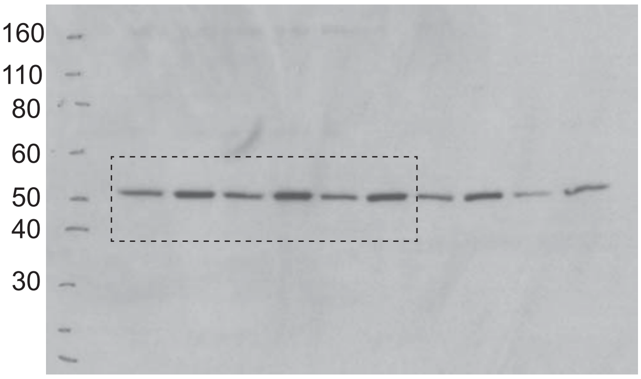

Figure 3A_MLKL

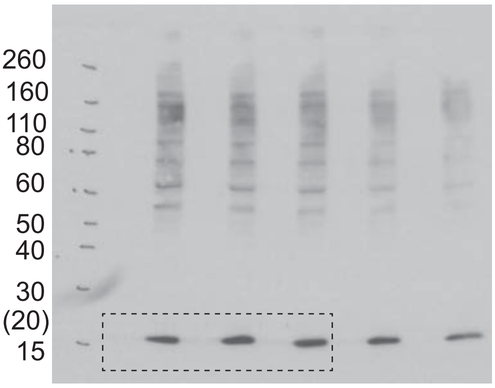

Figure 3A_ISG15

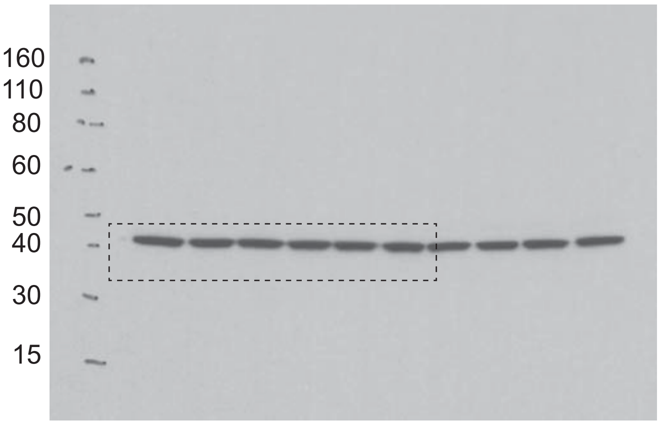

Figure 3A_ACTB

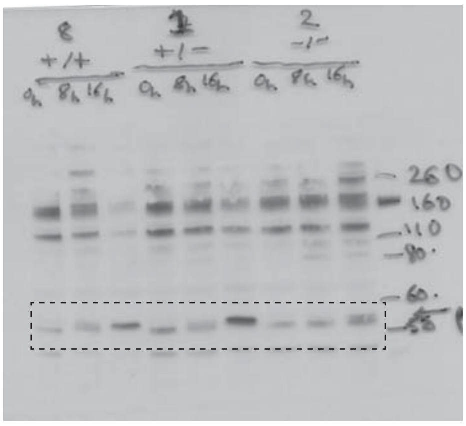

Figure 3D_P-MLKL

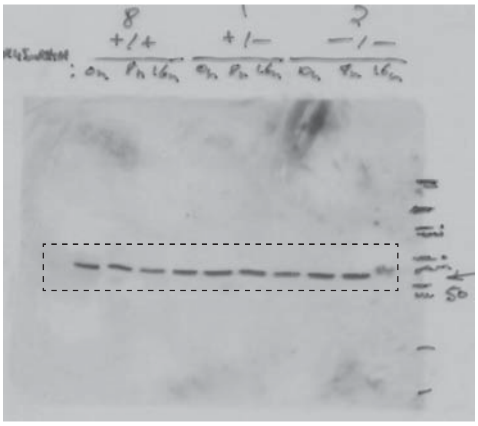

Figure 3D_ZBP1 MLKL

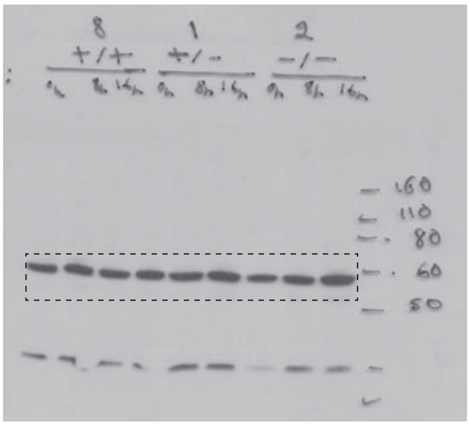

Figure 3D_ZBP1 (Zippy-1)

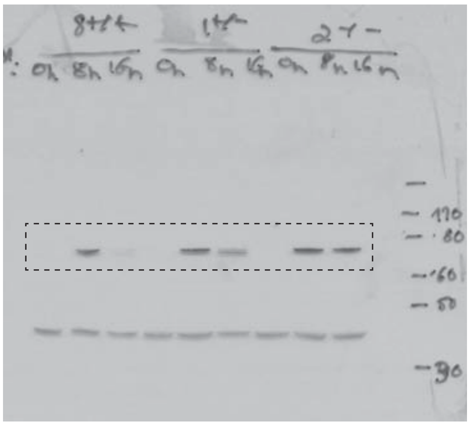

Figure 3D_IE1

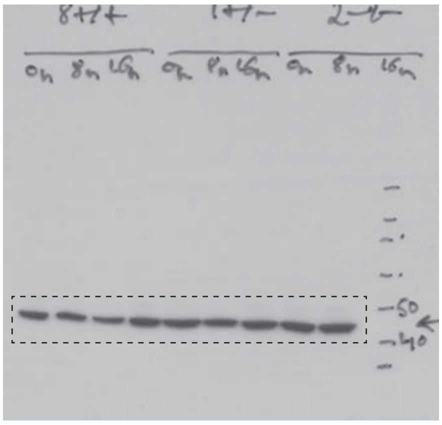

Figure 3D_ACTB

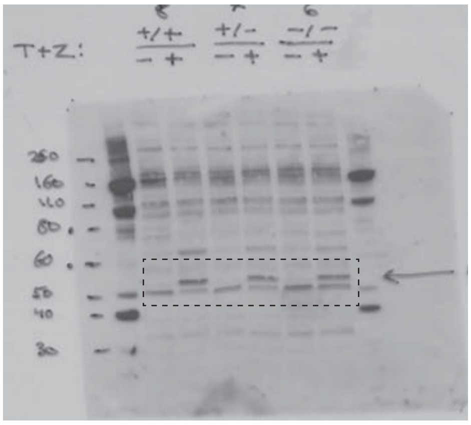

Figure 3E_P-MLKL

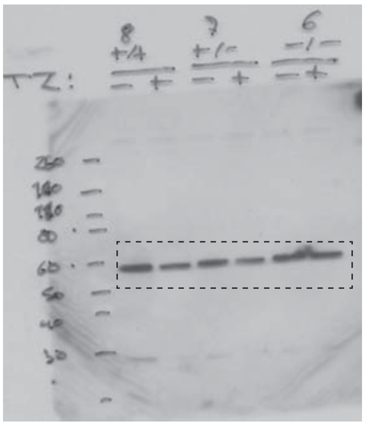

Figure 3E_ZBP1 (Zippy-1)

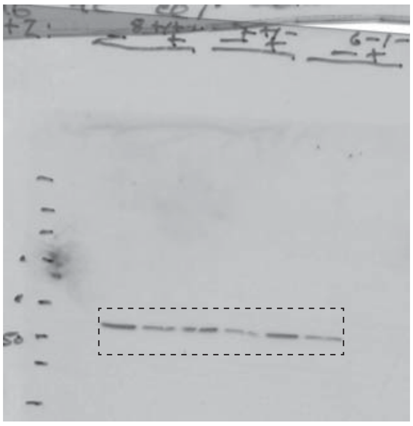

Figure 3E_MLKL

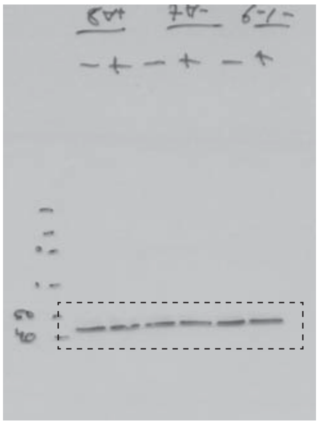

Figure 3E_ACTB
