# Supplementary material for: Sensing of viral and endogenous RNA by ZBP1/DAI induces necroptosis
Source: EMBO J. 2017 Jul 17;36(17):2529–43. doi: 10.15252/embj.201796476 (PMC5579359; doi:10.15252/embj.201796476)
Supplement: Supplementary file 9 — Source Data for Figure 5 [file EMBJ-36-2529-s007.pdf]

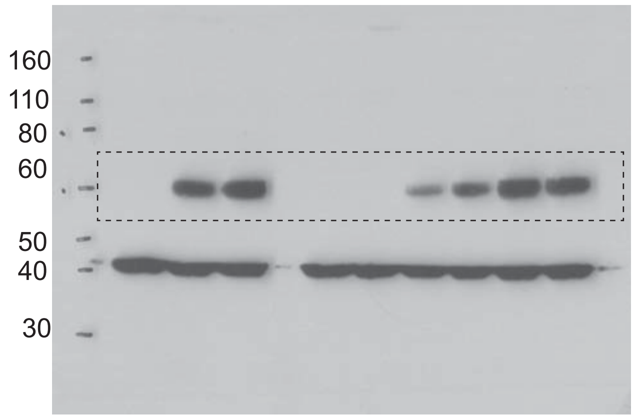

Figure 5C_FLAG (&ACTB)

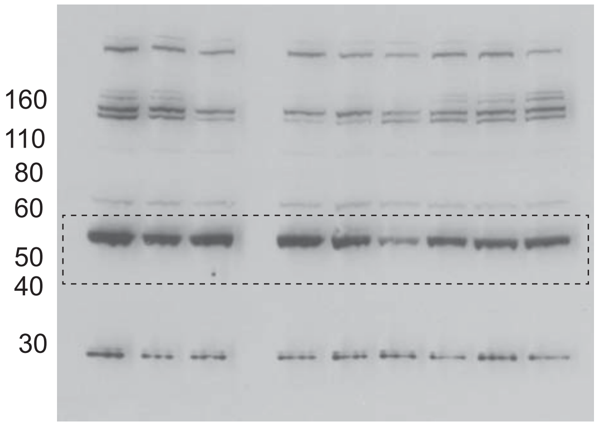

Figure 5C_RIPK3

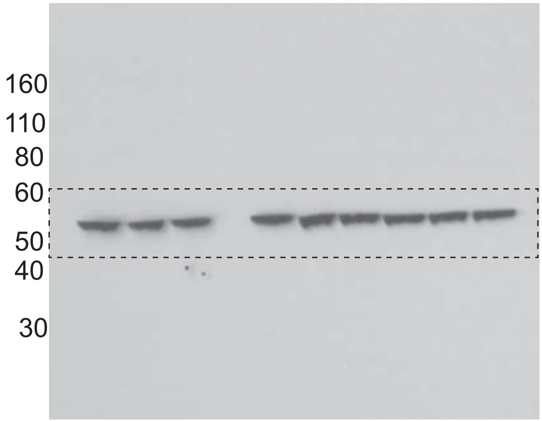

Figure 5C_MLKL

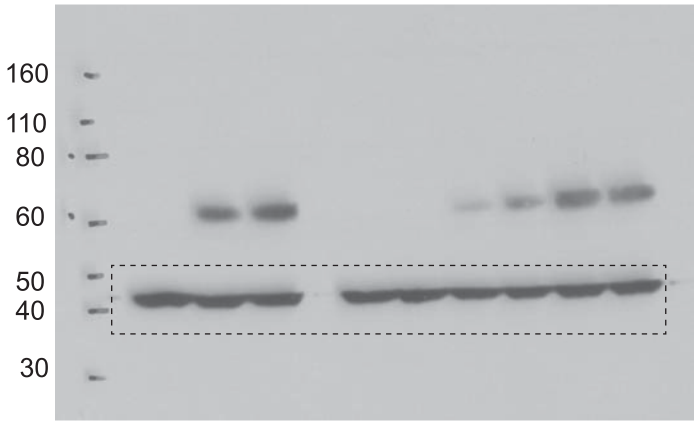

Figure 5C_(FLAG)&ACTB

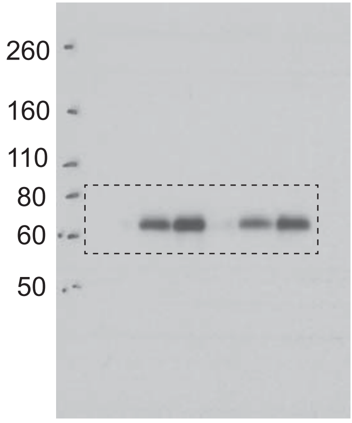

Figure 5F_FLAG

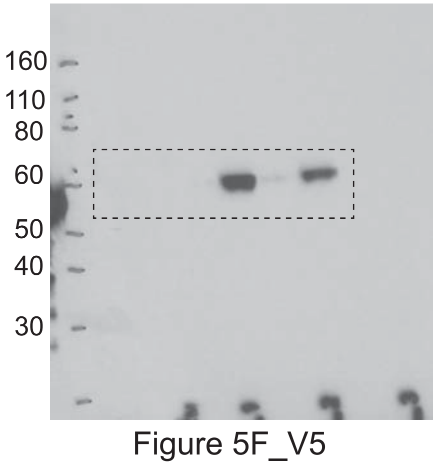

Figure 5F_V5

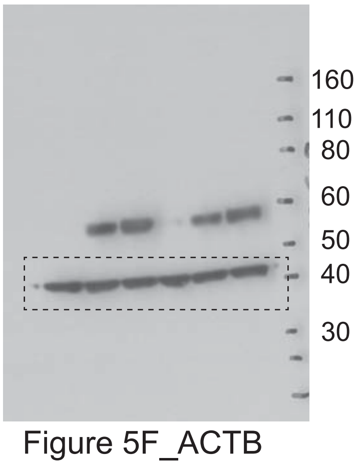

Figure 5F_ACTB
